# Staining patterns of PNA and UEA-I lectins in the postnatal developing male genital excurrent duct epithelium in mice

**DOI:** 10.5455/javar.2024.k801

**Published:** 2024-06-24

**Authors:** Md. Royhan Gofur, Kazushige Ogawa

**Affiliations:** 1 Department of Veterinary and Animal Sciences, University of Rajshahi, Rajshahi, Bangladesh; ^2^Laboratory of Veterinary Anatomy, Graduate School of Veterinary Science, Osaka Metropolitan University, Osaka, Japan

**Keywords:** PNA, UEA-I, postnatal development, excurrent duct epithelium, male, mice

## Abstract

**Objective:**

The research was aimed at determining the staining patterns of Peanut agglutinin (PNA) lectin and Ulex europaeus agglutinin I (UEA-I) lectin in postnatal developing (day 1 to 8 weeks) male genital excurrent duct epithelium in mice.

**Materials and Methods:**

Lectin staining was performed on testis and epididymis of 1-day-old and 1-, 2-, 3-, 4-, 5-, 6-, and 8-week-old ICR mice.

**Results:**

Rete testis epithelia were UEA-I- and PNA-negative at all postnatal ages. PNA lectin unclearly stained the epithelial apical surface in efferent ductules (ED) and ductus epididymis up to 2 weeks, while UEA-I was negative in those epithelia. By contrast, at 3 weeks of age, PNA and/or UEA-I turned to clearly label epithelia in the excurrent duct system. At 5 weeks, efferent ductules epithelium was UEA-I-negative but strongly PNA-positive; segment I was PNA-negative but faintly UEA-I positive; segment II was weakly PNA-positive but strongly UEA-I-positive; segment III was strongly-PNA- and UEA-I-positive; segment IV was weakly UEA-I-positive but strongly PNA-positive; and segment V was faintly UEA-I-positive but strongly PNA-positive. At 5 and 8 weeks (the adult), the staining patterns of PNA and UEA-I lectins were nearly identical. Moreover, an increasing staining intensity was observed in both lectins with the advancement of age until 5 weeks of age. Additionally, it was noted that, with the exception of segment I, the staining intensity of PNA gradually increased while that of UEA-I gradually decreased towards the distal section of the ductus epididymis.

**Conclusion:**

Lectin PNA and UEA-I staining, in conclusion, indicated epithelial segments in the male genital duct of mice from the postnatal developing age of 3 weeks, and up until 5 weeks, their staining patterns were nearly identical to those in adults.

## Introduction

During their transit through the extra-testicular excurrent duct system, mammalian spermatozoa undergo biochemical and physiological changes that enable them to acquire motility and fertilize, thereby becoming functionally mature [1,2]. Tissue-specific epithelia line the rete tesis, ductuli efferentes, ductus epididymidis, and ductus deferens, which make up the male genital excurrent duct system [3]. The rete testis, ductus epididymis, and efferent ductules develop from the testicular cords, mesonephric duct, and mesonephric tubules, respectively [4]. The rete testis and the ductus epididymis are connected by the efferent ductules [5]. Consequently, the junction at both ends of efferent ductules is assigned two developmental boundaries, and the epithelia of these tissues histologically maintain these developmental boundaries in adulthood [6–8]. The rete testis has a basic cuboidal epithelium lining it. When the epithelium transforms from a basic cuboidal shape to a basic columnar form, the ductuli efferentes start. The ductus epididymis is underdeveloped at birth, and the first few weeks after birth are a time of significant epididymal remodeling. At 5 weeks of age, the mouse epididymis acquires the histological and histochemical characteristics of an adult [9,10]. The head, body, and tail are three general divisions of mature epididymis. Histologically and histochemically, it can be further segmented based on how differently glycoconjugates or sugar chains are expressed in the epithelium [11,12]. Different gene expression profiles in each segment ensure unique epididymal functions that are crucial to the various stages of sperm maturation [13,14].

Lectins are proteins that bind carbohydrates and serve as recognition molecules in a range of biological processes, including interactions between molecules and between cells [15,16]. The discovery that lectins are incredibly useful tools for studying cell surface sugars and evaluating their roles in cell growth and differentiation, interactions between cells and their environment, and a range of pathological processes sparked an increased interest in the protein [17,18]. Because lectins have a unique affinity for a particular sugar, they bind precisely to the carbohydrate residues of glycoconjugates. Sugar chains are known to have a variety of physiological roles in the body and can attach not just to other sugars but also to lipids, proteins, and other tiny particles. More recently, lectins coupled to probes with fluorescent molecules or to enzyme biomarkers like horseradish peroxidase have been utilized to cytochemically localize macromolecules rich in carbohydrates [19]. The ability of diverse lectins to exhibit specificity towards distinct sugar sequences or sugar residues facilitates the classification of complex carbohydrates based on the kinds of internal or terminal sugars or sugar links they contain [20]. Thus, lectins can be used as histochemical probes to identify glycomolecules “*in situ*” in tissues and cells and to characterize cell populations within an organ in various species. Lectins are also used in the identification of segments of segmented organs like the nephron [21,22].

Staining patterns of several lectins in the epithelia of male genital organs have been studied in various animals [11,12,23–29]. We, therefore, employed fluorescence labeling of a mixture of peanut agglutinin (PNA) and ulex europaeus agglutinin I (UEA-I) to differentiate the epithelial segments of the male genital excurrent duct in ICR mice and to determine the staining patterns of PNA and UEA-I lectins in the male genital excurrent duct epithelium during postnatal development (days 1–8 weeks), as well as when, throughout the postnatal development of mice, the male genital excurrent duct epithelium exhibits the identical segment-specific feature as observed in adult epithelia with regard to the lectin affinity.

## Materials and Methods

### Animals and ethical approval

For histochemical investigations, ICR male mice aged 1 day, 1, 2, 3, 4, 5, 6, and 8 weeks, housed in conventional housing and food settings, were utilized. The Animal Research Committee (ARC) of Osaka Metropolitan University approved the protocols for animal experimentation (approval number: 29−12).

### Antibodies and lectins

A rabbit polyclonal antibody (ab5694) against alpha-smooth muscle actin (α-SMA) was procured from Abcam (Cambridge, UK). Alexa Fluor 568-conjugated donkey anti-rabbit immunoglobulin G (IgG) was purchased from Molecular Probes, Inc. (Eugene, OR, USA). Fluorescein isothiocyanate-conjugated peanut agglutinin lectin (FITC-PNA) lectin was obtained from Sigma-Aldrich (St. Louis, MO, USA), and rhodamine-conjugated Ulex Europaeus Agglutinin I lectin (Rh-UEA-I) lectin was procured from Vector Laboratories (Burlingame, CA, USA).

### Lectin fluorescence and immunofluorescence staining

The tissue samples were preserved in formalin (10%) at 4°C for various times at the various ages displayed in Table 1. Following phosphate buffered saline (PBS) washing, submerged for 3.5 h overnight in a 30% sucrose solution in PBS (Table 1), and then embedded in the optimal cutting temperature compound. Next, fluorescent staining was applied to cryostat sections that were 5 μm thick.

Lectin fluorescence staining was carried out in accordance with earlier instructions [30]. Briefly, cryostat sections were treated for 0.5 h at 32°C with 5 µg/ml FITC-PNA and/or 1 µg/ml Rh-UEA-I after being incubated in a 1% bovine serum albumin (BSA) in a PBS humid chamber at 25°C for 0.5 h. Later, the sections underwent PBS washing, PermaFluor mounting, and fluorescence microscopy photography.

**Table 1.** Tissue sample fixation protocols for lectin staining.

Mice age	Fixation time	Washing time	Soaking time in 30% sucrose solution	Amount of fixative/tissue
1d	2 h	1 h	3.5 h	1 ml
1w	2 h	1 h	4.5 h	1 ml
2w	3.5 h	1 h	7 h	10 ml
3w—8w	4 h	1 h	Overnight	10 ml

A combination of α-SMA and FITC-PNA immunofluorescence labeling was accomplished as earlier defined [30]. In summary, sections (cryostat) were treated with 1% BSA in PBS in a humid chamber and then incubated with the primary antibody at a concentration of 1:400 α-SMA at 32°C for 1.5 h. Following a PBS wash, incubate the selected sections for 0.5 h at 32°C with a combination of 5 µg/ml of FITC-PNA and 5 µg/ml of Alexa Fluor 568-conjugated donkey anti-rabbit IgG. Subsequently, the sections underwent PBS washing, PermaFluor mounting, and fluorescence microscopy photography. By incubating adult mice without antibodies, either primary or secondary, the staining specificity was confirmed.

## Results

The histological segments of the genital duct system in males, which include rete testis, efferent ductules, segments I–III in the head, segments IV in the body, and V in the tail of the epididymis, were demonstrated in our previous study to exhibit specific staining patterns of lectins in the epithelia of adult mice [7]. Thus, we aimed to determine the lectin (PNA and UEA-I) staining patterns in male genital excurrent duct epithelium during postnatal development (day 1 to 8 weeks), as well as regarding lectin affinity, when, throughout the postnatal development of mice, the male genital excurrent duct epithelium displays the same feature exclusive to a particular segment as mature epithelia.

**Figure 1.**
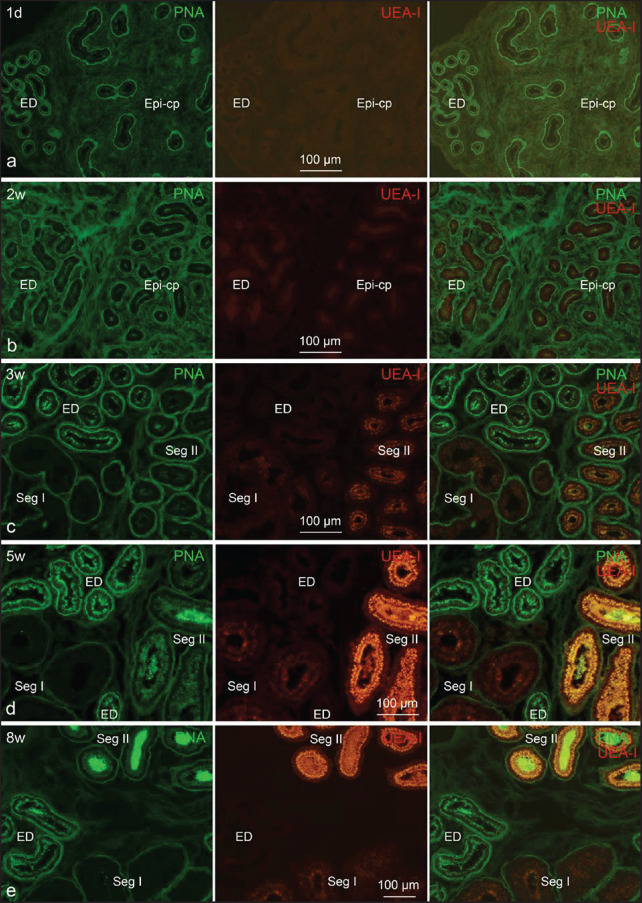
Lectin fluorescence micrographs showing PNA and UEA-I lectin staining patterns in the male genital excurrent duct epithelium during the postnatal development. Sections were stained with the indicated lectins. (a-b) PNA lectin faintly and unclearly stain the apical surface of epithelium of the efferent ductules (ED) and ductus epididymis (Epi-cp) up to 2 weeks whereas UEA-I staining was clearly negative. (c) At 3 weeks (3w), epithelia of the ED are weakly PNA-positive and UEA-I-negative; epithelia of the segment I (Seg I) are PNA-negative and faintly UEA-I-positive; epithelia of the segment II (Seg II) are faintly PNA-positive and weakly UEA-I-positive. (d-e) At 5 weeks (5w), the lectin staining patterns are almost the same as those in the adult (8 weeks, 8w): epithelia of the ED are strongly PNA-positive but UEA-I-negative; epithelia of the se Seg I are PNA-negative and faintly UEA-I-positive; epithelia of the Seg II are weakly PNA-positive and strongly UEA-I-positive. Both PNA and UEA-I stained the spermatozoa in the lumen of the ductus epididymis.

The epithelium of the rete testis was PNA- and UEA-I-negative at all postnatal ages. PNA lectin faintly and unclearly stain the epithelial apical surface of efferent ductules and ductus epididymis for up to 2 weeks, whereas UEA-I staining was clearly negative (Fig. 1a–b). In contrast, epithelia of the excurrent duct abruptly became stained by both studied lectins at the age of 3 weeks: efferent ductules epithelium was UEA-I-negative but weakly PNA-positive; segment I epithelium was faintly UEA-I-positive but PNA-negative; segment II and III epithelia were faintly PNA-positive and weakly UEA-I-positive; segment IV epithelium was faintly PNA- and UEA-I-positive; segment V epithelium was UEA-I-negative but weakly PNA-positive (Fig. 1c). When the lectin staining patterns were examined at 5 weeks, they were nearly identical to those observed at the age of 8 weeks (the adult): efferent ductules epithelium was UEA-I-negative but strongly PNA-positive; segment I epithelium was PNA-negative and faintly UEA-I-positive; segment II epithelium was weakly PNA-positive, but strongly UEA-I-positive; segment III epithelium was strongly PNA- and UEA-I-positive; segment IV epithelium was strongly PNA-positive, but weakly UEA-I-positive; segment V epithelium was faintly UEA-I-positive, but strongly PNA-positive (Fig. 1d). Thus, up until 5 weeks of age, the staining patterns of both lectins were nearly identical to those in the adult (Fig. 1e).

**Figure 2.**
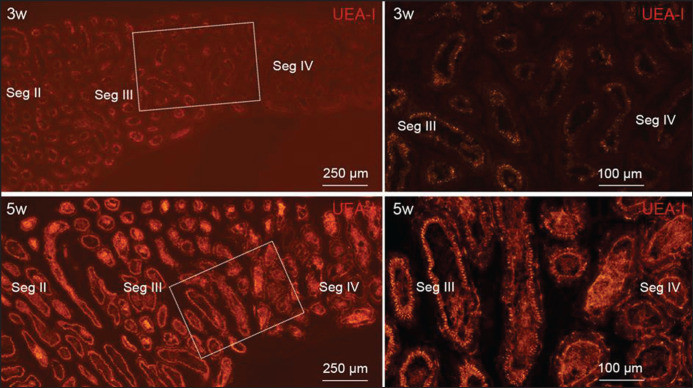
Fluorescence micrographs showing staining pattern of UEA-I lectin in epithelium of different segment of ductus epididymis at 3 and 5 weeks of postnatal age. Sections were stained with the UEA-I lectin. At 3 weeks of age, epithelia of the segment II and III are weakly UEA-I-positive whereas those of the segment IV are faintly UEA-I-positive, whereas at 5 weeks of age, epithelia of the segment II and III are strongly UEA-I-positive whereas those of the segment IV are weakly UEA-I-positive. Seg, segment.

**Figure 3.**
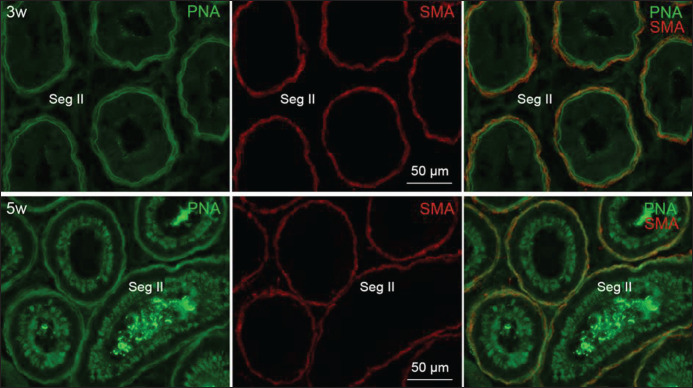
Fluorescence micrographs showing staining pattern of PNA lectin in epithelium and α-SMA-positive smooth muscle cells surrounding the ductus epididymis at 3 and 5 weeks of postnatal age. Epithelia of the segment II are faintly PNA-positive at 3 weeks whereas weakly PNA-positive at 5 weeks of age. PNA stained the α-SMA-positive smooth muscle cells at both ages. Seg, segment.

**Figure 4.**
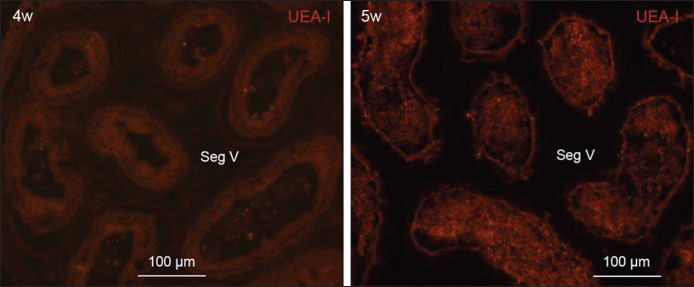
Fluorescence micrographs showing staining pattern of UEA-I lectin in epithelium of segment V of ductus epididymis at 4 and 5 weeks of postnatal age. Epithelia of the segment V are UEA-I-negative at 4 weeks whereas faintly UEA-I-positive at 5 weeks of age. Seg, segment.

We found a clear differential picture of the staining intensity of the studied lectins in terms of postnatal developing age and different tissues/segments of the male genital excurrent duct system. An increasing staining intensity was observed in both PNA lectin and UEA-I lectin with the advancement of age until 5 weeks of age (Fig. 1a–e, Figs. 2–4, and Table 2). Moreover, it was noted that, with the exception of segment I, the staining intensity of PNA gradually increased while that of UEA-I gradually decreased towards the distal section of the ductus epididymis (Fig. 2 and Table 2).

Additionally, PNA stained the flattened smooth muscle cells (α-SMA-positive) adjoining the ductus epididymis and efferent ductules at all postnatal ages (Fig. 3). Both lectins stained substances in the lumen of excurrent ductules/ducts in early postnatal ages and spermatozoa from the age of 5 weeks. The staining patterns of studied lectins in the epithelium of male genital ductal segments at varying postnatal ages are compiled in Table 2.

## Discussion

Using lectin histochemistry, we revealed PNA and UEA-I staining patterns in the male genital excurrent duct epithelium during postnatal development. As far as current research indicates, this is the first study to evaluate the staining affinities of UEA-I and PNA lectins in male genital excurrent duct epithelia during growth after birth. We found a clear differential picture of the staining intensity of the studied lectins in terms of postnatal developing ages. The epithelium of the excurrent duct up to 2 weeks showed no (UEA-I) or minor (PNA) affinity to lectins, indicating immature/undifferentiated epithelial cells of the male genital excurrent duct probably have no or minor affinity to lectins. A similar discrepancy in the affinity of several lectins was detected in the rete testis epithelium between immature and sexually mature horses. The immature epithelium has comparatively lower affinity than the mature epithelium [31]. The ductus epididymis is underdeveloped at birth, and during the first few weeks after birth, epithelial cells, which are populated by numerous undifferentiated or immature columnar tall epithelial cells and a few small mitotic cells, routinely go through mitosis. Subsequently, the ductus epididymis becomes functional as epithelial cells start to differentiate or mature [9,32–33]. According to the current research, variations in lectin-binding patterns across various postnatal ages correspond to variations in the state of epithelial maturation (immature or mature).

An increasing staining intensity was observed in both PNA lectin and UEA-I lectin with the advancement of age from 3 weeks to 5 weeks of age, and then it remained almost constant. The current findings demonstrated that the lectin staining patterns were nearly identical to those in 8 weeks (the adult) until 5 weeks of postnatal age, suggesting that the developing postnatal male genital excurrent duct epithelium displayed the same segment-specific characteristics as observed in the epithelia of adult mice with regard to lectin staining at this age. The current results are consistent with the research stating that around 5 weeks of age in mice, the epididymis attains the histological and histochemical characteristics defined as the adult [9–10].

**Table 2.** PNA and UEA-I staining patterns in male genital excurrent duct epithelium in mice during the postnatal development.

Epithelium		PNA					UEA-I			
		1d−2w	3w	4w	5w−8w		1d−2w	3w	4w	5w−8w
Rete testis		−	−	−	−		−	−	−	−
Efferent ductules		−/±	+	++	++		−	−	−	−
Epididymis	Seg I	−/±	−	−	−		−	±	±	±
	Seg II	−/±	±	±	+		−	+	++	++
	Seg III	−/±	±	++	++		−	+	++	++
	Seg IV	−/±	±	++	++		−	±	+/±	+
	Seg V	−/±	+	++	++		−	−	−	±

Seg, Segment; −, negative; ±, faint stain; +, weak stain; ++, strong stain.

Our findings showed that staining patterns of a particular lectin in the epithelia vary among different regions of the male duct system in mice. The variations in lectin-binding patterns correspond to variations in their functions in their respective regions or segments. Moreover, PNA lectin and UEA-I lectin staining patterns were nearly vice versa in different epithelial segments of the male genital excurrent duct system. The staining intensity of PNA was gradually increased, whereas the staining intensity of UEA-I was gradually decreased towards the distal part of the ductus epididymis, except for segment-I. This implies that variable sugar chains are introduced to distinct epithelial locations in the male genital duct system.

There were some contrasts in the staining patterns of PNA and UEA-I between those observed in this work and those found in other species, such as horses [31], alpacas [34], etc. For example, UEA-I showed no reaction to the epithelium of efferent dyctules in this study in mice but showed a positive reaction in horses. PNA showed a positive reaction in the epithelium of efferent dyctules in this study in mice, but no reaction was found in this tissue in alpaca. These discrepancies are most likely the result of variations in the fixation procedure, the sensitivity of the detection techniques, or species variations.

## Conclusion

Male genital excurrent duct epithelium showed different lectin staining patterns that are segment-specific, suggesting a great level of partitioning for absorptive and secretory processes in the excurrent duct, which may be crucial for sperm maturation. Immature epithelial cells have no or minor affinity for studied lectins, but staining intensity gradually increased with age until 5 weeks of age, when the staining pattern was identical to the adult tissue, indicating the postnatal developing male genital excurrent duct epithelium showed a similar segment-specific characteristic of lectin staining in mice at this age that is observed in adult epithelia. Moreover, due to segment-specific staining patterns, the UEA-I and PNA lectin s combination may be used to recognize the epithelial subdivisions of the male genital duct at postnatal 3 weeks of age in mice.

## List of Abbreviations

μm, micrometer; µg, microgram; mL, milliliter; h, hour; °C, degree Celsius; IgG, Immunoglobulin G; BSA, *bovine serum albumin*; OCT, optimal cutting temperature; PBS, phosphate buffered saline; *PNA,* Peanut agglutinin; *UEA-I,* Ulex europaeus agglutinin I; α-SMA, alpha smooth muscle actin; FITC-PNA, Fluorescein isothiocyanate-conjugated peanut agglutinin lectin; Rh-UEA-I, rhodamine-conjugated Ulex Europaeus Agglutinin I lectin;.
